# Development of a method for reconstruction of crowded NMR spectra from undersampled time-domain data

**DOI:** 10.1007/s10858-015-9908-9

**Published:** 2015-02-13

**Authors:** Takumi Ueda, Chie Yoshiura, Masahiko Matsumoto, Yutaka Kofuku, Junya Okude, Keita Kondo, Yutaro Shiraishi, Koh Takeuchi, Ichio Shimada

**Affiliations:** 1 Graduate School of Pharmaceutical Sciences, The University of Tokyo, Hongo, Bunkyo-ku, Tokyo, 113-0033 Japan; 2 Precursory Research for Embryonic Science and Technology, Japan Science and Technology Agency, Chiyoda-ku, Tokyo, 102-0075 Japan; 3 Japan Biological Informatics Consortium, Aomi, Koto-ku, Tokyo, 135-8073 Japan; 4 Molecular Profiling Research Center, National Institute of Advanced Industrial Science and Technology, Aomi, Koto-ku, Tokyo, 135-0064 Japan

**Keywords:** Sparse sampling, Non-uniform sampling, Transferred cross-saturation, Membrane proteins

## Abstract

NMR is a unique methodology for obtaining information about the conformational dynamics of proteins in heterogeneous biomolecular systems. In various NMR methods, such as transferred cross-saturation, relaxation dispersion, and paramagnetic relaxation enhancement experiments, fast determination of the signal intensity ratios in the NMR spectra with high accuracy is required for analyses of targets with low yields and stabilities. However, conventional methods for the reconstruction of spectra from undersampled time-domain data, such as linear prediction, spectroscopy with integration of frequency and time domain, and analysis of Fourier, and compressed sensing were not effective for the accurate determination of the signal intensity ratios of the crowded two-dimensional spectra of proteins. Here, we developed an NMR spectra reconstruction method, “conservation of experimental data in analysis of Fourier” (Co-ANAFOR), to reconstruct the crowded spectra from the undersampled time-domain data. The number of sampling points required for the transferred cross-saturation experiments between membrane proteins, photosystem I and cytochrome *b*_*6*_ *f*, and their ligand, plastocyanin, with Co-ANAFOR was half of that needed for linear prediction, and the peak height reduction ratios of the spectra reconstructed from truncated time-domain data by Co-ANAFOR were more accurate than those reconstructed from non-uniformly sampled data by compressed sensing.

**Electronic supplementary material:**

The online version of this article (doi:10.1007/s10858-015-9908-9) contains supplementary material, which is available to authorized users.

## Introduction

Living cells have quite inhomogeneous components, and contain an abundance of complex architectures existing in both solid and liquid phases. Therefore, structural analyses of proteins under physiological conditions are prerequisite to clarify their functional mechanisms. In addition, accumulating evidence has suggested that proteins are quite dynamic and interconvert between multiple conformations in equilibrium. NMR methods, such as transferred cross-saturation (TCS) (Nakanishi et al. 2002; Ueda et al. 2014), residual dipolar couplings (Tjandra and Bax 1997; Tolman and Ruan 2006; Ban et al. 2013), relaxation dispersion (Palmer 2004; Baldwin and Kay 2009), and paramagnetic relaxation enhancement experiments (Iwahara and Clore 2006; Tang et al. 2006; Clore and Iwahara 2009; Clore 2014) are useful for obtaining information about the conformational dynamics of proteins in heterogeneous systems. In these NMR experiments, fast determination of the signal intensity ratios in the two-dimensional NMR spectra with high accuracy is required for the analyses of targets with low yields and stabilities.

High resolution NMR spectra can be reconstructed from undersampled time-domain data by inserting calculated data into the unobserved data point region, because many multidimensional NMR spectra are sparse: the resonances occupy only a small fraction of the spectra (Kazimierczuk and Orekhov 2011; Mayzel et al. 2014a). The reconstruction of the spectra from undersampled data, as well as enhancement of longitudinal relaxation (Hiller et al. 2005; Schanda et al. 2006; Theillet et al. 2011) and parallel acquisition (Tal et al. 2009), enables fast multidimensional NMR data acquisition. In addition to the linear prediction (LP) method (Barkhuijsen et al. 1985a; Schussheim and Cowburn 1987), a variety of reconstruction methods have been developed (Barkhuijsen et al. 1985b; Barna and Laue 1987; Hoch 1989; Chen et al. 2000; Stern et al. 2002; Kim and Szyperski 2003; Freeman and Kupce 2004; Kupce and Freeman 2004; Jaravine et al. 2006; Hyberts et al. 2007, 2010, 2012, 2014; Hiller et al. 2008; Mobli and Hoch 2008; Kazimierczuk and Orekhov 2011; Shrot and Frydman 2011; Mobli and Hoch 2014; Qu et al. 2015). Collecting long evolution time points by non-uniform or radial sampling is reportedly useful for managing the decreased amount of time-domain data in several reported reconstruction methods (Stern et al. 2002), such as compressed sensing (CS) (Kazimierczuk and Orekhov 2011), which is reportedly orders of magnitude faster than forward maximum entropy reconstruction (Hyberts et al. 2012), and these methods were applied to the reconstruction of the multidimensional NOESY spectra, which have weak signals and strong diagonal peaks (Stern et al. 2002; Hyberts et al. 2009, 2012; Orekhov and Jaravine 2011; Bostock et al. 2012).

In most multidimensional NMR experiments with biomolecules, additional simpler and more sensitive NMR data, such as ^1^H–^15^N heteronuclear single quantum coherence (HSQC) or transverse relaxation optimization spectroscopy (TROSY) spectra, are usually recorded before and after the multidimensional NMR measurements, for the preliminary sample characterization. The chemical shifts and linewidths of these data, which were previously referred to as “guide-FIDs” (Bodart et al. 2002; Lippens et al. 2003), are, in principle, identical to those in the multidimensional NMR spectra. In several reconstruction methods, such as technique for importing greater evolution resolution (McGeorge et al. 1997), analysis of Fourier (ANAFOR) (Bodart et al. 2002; Lippens et al. 2003), co-processing of several spectra by multi-dimensional decomposition (Orekhov and Jaravine 2011), hyperdimensional NMR spectroscopy (Kupce and Freeman 2006; Jaravine et al. 2008; Orekhov and Jaravine 2011; Mayzel et al. 2014b), spectroscopy with integration of frequency and time domain (SIFT) (Matsuki et al. 2009, 2011), and other methods (Manassen et al. 1987; Schuyler et al. 2011), the chemical shifts and relaxation rates determined by the guide-FIDs are utilized to increase the accuracy in the reconstruction of the multidimensional NMR spectra, and spectra in the relaxation dispersion experiments were reportedly reconstructed by SIFT (Matsuki et al. 2011).

Uniformly sampled truncated data contains larger amounts of the data with small evolution times, in which the signal-to-noise ratio (S/N) is high, than data with a sinusoidal weighted Poisson-Gap distribution, which are reportedly effective in the cases of CS (Hyberts et al. 2012). Therefore, it is possible that uniform truncated sampling will be effective for experiments which require accurate signal intensity ratios. However, the comparison of non-uniform sampling with truncated uniform sampling was not performed in these experiments.

Here, we developed a multidimensional NMR spectra reconstruction method, based on ANAFOR (Bodart et al. 2002; Lippens et al. 2003), which was applicable to the reconstruction of crowded spectra from a small amount of truncated time domain data. The developed method was evaluated, concerning the accuracy of the peak height ratios of the reconstructed spectra.

## Experimental section

### Processing of the experimentally observed data of EIN, plastocyanin, and ubiquitin

The width of the bright region in SIFT was set to 54 Hz, and the iteration was repeated 50 times. ^1^H–^15^N HSQC or ^1^H–^15^N TROSY spectra recorded before the truncated data measurements were utilized as guide-FIDs in the reconstruction by SIFT, ANAFOR and Co-ANAFOR, unless otherwise stated. The Tikhonov regularization factor (See Electronic Supplementary Material for details) and the linewidth of the signals in the directly observed dimension, which were utilized in the reconstruction by Co-ANAFOR, were set to 0.01 and ±0.02 ppm, respectively, unless otherwise stated. The relaxation rates of the signals, utilized for the calculation of the inserted data, were uniformly set to the reciprocal of the maximum evolution time after the insertion of the calculated data, unless otherwise stated. Non-uniform sampling schedules were prepared by Schedule Generator Version 3.0 (http://gwagner.med.harvard.edu/intranet/hmsIST/gensched_new.html) (Hyberts et al. 2010). Sinusoidal weight was set to 2. Reconstruction of the spectra by CS using iterative soft thresholding were performed by hmsIST (http://gwagner.med.harvard.edu/intranet/hmsIST/index.html) (Hyberts et al. 2012). Number of iteration was set to 400. Non-uniformly sampled data were generated from uniformly sampled data and the sampling schedules by an in-house developed program. The square sine with a 90° phase shift window function was manipulated after the insertion, unless otherwise stated.

Uniformly deuterated and ^15^N-labeled EIN and non-labeled histidine-containing phosphocarrier protein (HPr) were prepared as reported previously (Suh et al. 2008). The ^1^H–^15^N TROSY experiments with EIN were performed at 303 K, using 0.5 mM [ul-^2^H, ^15^N] EIN, 1 mM non-labeled HPr, pH 6.0, and 80 % D_2_O, with a Bruker Avance 600 spectrometer equipped with a cryogenic probe. The maximum evolution times in the ^1^H and ^15^N dimensions after the reconstruction were set to 106.5 and 65.8 ms, respectively. The linewidth of the signals in the directly observed dimension was set to ±0.05 ppm. The number of coefficients in LP was set to 16.

The TCS spectra of spinach plastocyanin and thylakoid vesicles were recorded as described previously (Ueda et al. 2012). The maximum evolution times in the ^1^H and ^15^N dimensions after the insertion of the time-domain data were set to 142.5 and 20.3 ms, respectively. TCS spectra of spinach plastocyanin and solubilized photosystem I were utilized as the guide-FIDs in Co-ANAFOR (Ueda et al. 2012). The relaxation rate of the signals, utilized for the calculation of the inserted data, was set to 6.3 s^−1^. The number of coefficients in LP was set to 8.

^1^H–^15^N HSQC experiments of [ul-^15^N] plastocyanin in the presence or absence of 400 μM tetralysine were performed at 303 K, using 40 μM plastocyanin, 20 mM Bis–Tris, pH 6.0, 2 mM ascorbic acid, and 10 % D_2_O, with a Bruker Avance 500 spectrometer equipped with a cryogenic probe. The maximum evolution times in the ^1^H and ^15^N dimensions after the insertion of the time-domain data were set to 127.8 and 37.1 ms, respectively. The relaxation rate of the signals, utilized for the calculation of the inserted data, was set to 9.3 s^−1^.

Uniformly ^13^C- and ^15^N-labeled ubiquitin were prepared as reported previously (Moriya et al. 2009). The ^15^N-edited TROSY–NOESY experiments of ubiquitin were performed at 303 K, using 1 mM [ul-^13^C, ^15^N] in 20 mM sodium phosphate, pH 6.0, 40 mM NaCl, and 10 % D_2_O, with a Bruker Avance 600 spectrometer. The maximum evolution times in the F1 (^1^H), F2 (^15^N), and F3 (^1^H) dimensions before the insertion of the time-domain data were set to 7.56, 29.2, and 154.8 ms, respectively. The Tikhonov regularization factor was set to 0.1.

The programs for the reconstruction by the developed method are freely available on internet (http://ishimada.f.u-tokyo.ac.jp/public_html/reconstruction).

## Results

### Comparison of the available NMR spectra reconstruction methods

**Fig. 1:**
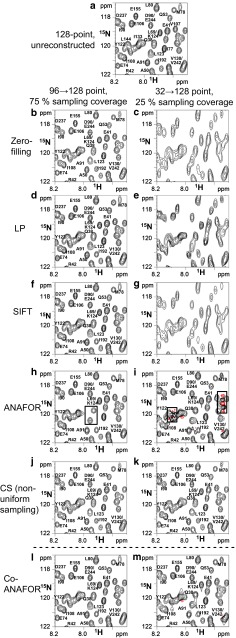
Comparison of the reconstruction methods using experimentally observed [^1^H, ^15^N] TROSY spectra at 14.1 T for [ul-^2^H,^15^N] EIN in complex with non-labeled HPr. **a** is the 128-point spectrum without reconstruction, and **b**–**k** are spectra reconstructed from 96-point (**b**, **d**, **f**, **h**, **j**) or 32-point (**c**, **e**, **g**, **i**, **k**) time-domain data, by zero-filling (**b**, **c**), LP (**d**, **e**), SIFT (**f**, **g**), ANAFOR (**h**, **i**), CS (**j**, **k**), or Co-ANAFOR (**l**, **m**). In **j** and **k**, non-uniformly sampled data were utilized for the reconstruction. Negative contours are colored *red*. In **h** and **i**, the resonances with *irregular lineshapes* are enclosed in *boxes*

To evaluate the NMR spectra reconstruction methods, ^1^H–^15^N TROSY spectra of the N-terminal domain of enzyme I (EIN) (Fig. 1a) were reconstructed from the truncated experimentally observed time-domain data, by zero-filling or inserting the data calculated by LP, SIFT, and ANAFOR (Fig. 1b–i). The spectra were also reconstructed from the non-uniformly sampled experimentally observed time domain data by inserting the data calculated by CS, which was not developed for the reconstruction of truncated data (Fig. 1j, k). The ratios of the numbers of data before and after the insertion of the data, which were previously referred to as “sampling coverage” (Schuyler et al. 2011), were set to 25 and 75 %, respectively.

The spectra with 75 % sampling coverage reconstructed by LP, SIFT, or CS (Fig. 1d, f, j) were almost identical to the unreconstructed spectra (Fig. 1a), whereas the lineshapes and intensities of the relatively broad resonances in the spectra reconstructed by ANAFOR (Fig. 1h) were significantly different from those of the unreconstructed spectra. These artifacts in ANAFOR are due to the differences between the linewidths and/or chemical shifts determined by the guide-FIDs and those of the signals in the truncated data, and these differences were previously referred to as “misinformation” (Bodart et al. 2002).

In the cases of 25 % sampling coverage, the resolutions of the spectra reconstructed by LP or SIFT were lower than that of the unreconstructed spectra (Fig. 1a, e, g), whereas the spectra reconstructed by CS were similar to the unreconstructed spectra (Fig. 1k). Although the resolution of the spectra reconstructed by ANAFOR was similar to that of the unreconstructed spectra, strong artifacts were observed in the crowded regions (Fig. 1a, i). The artifacts are derived from the instabilities of the least-squares method in the cases with large numbers of signals, relative to the number of truncated time-domain data.

### Development of the reconstruction method based on ANAFOR

To avoid the aforementioned problems with ANAFOR, we improved the original ANAFOR method as follows (See Electronic Supplementary Material for details). Firstly, the truncated time-domain data, which were replaced with the calculated data in ANAFOR, were conserved and the calculated data were inserted exclusively into the unrecorded data point region, to minimize the effects of the chemical shift and linewidth misinformation. Secondly, Tikhonov regularization (or ridge regression) (Tychonoff and Arsenin 1977), which is utilized in the multi-dimensional decomposition method (Luan et al. 2005), was applied to the determination of the signal intensities, because it reportedly increases the stability of the solution of the least-square methods. Hereafter, the developed reconstruction method is referred to as “conservation of experimental data in analysis of Fourier (Co-ANAFOR)”.

In the EIN spectra reconstructed by Co-ANAFOR with 75 % sampling coverage, the lineshapes were almost identical to those of the unreconstructed spectra (Fig. 1a, l), suggesting that the effect of the misinformation was decreased in Co-ANAFOR, by the conservation of the truncated time-domain data. In addition, the artifacts in the crowded region of the EIN spectra reconstructed by ANAFOR with 25 % sampling coverage (Fig. 1i) were markedly diminished in the spectra reconstructed by Co-ANAFOR (Fig. 1m), indicating that the solutions of the least-square methods were stabilized by the Tikhonov regularization in Co-ANAFOR.

### Evaluation of the developed method using synthetic data

**Fig. 2:**
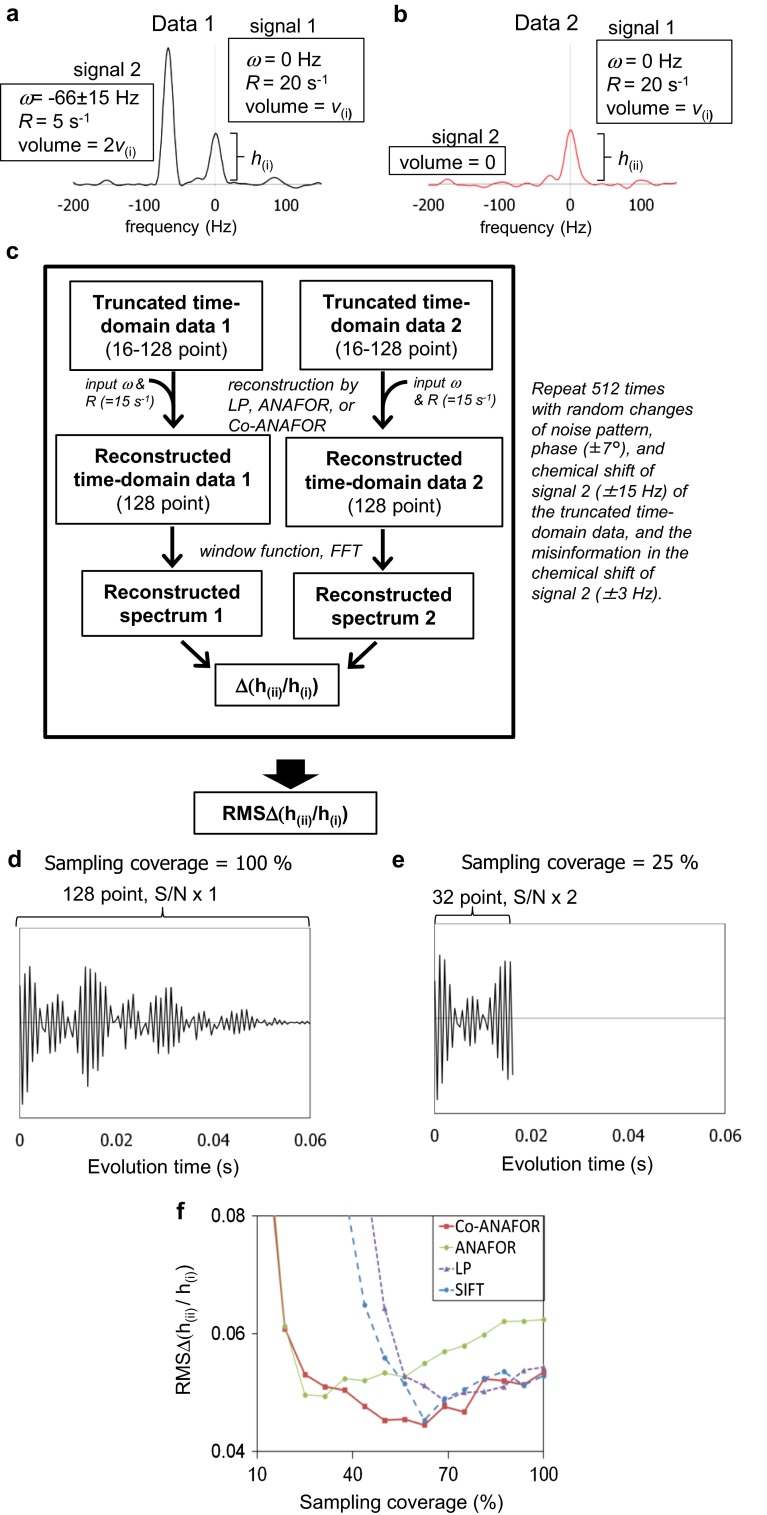
Evaluation of the reconstruction methods using synthetic data with noise. **a** and **b** are spectra of 128-point synthetic data 1 (**a**) and data 2 (**b**) without reconstruction. Data 1 are composed of signals 1 and 2, which are separated from each other by 66 Hz. In data 2, the peak volume of signal 2 is zero, whereas the chemical shift, relaxation rate, and peak volume of the signal 1 are the same as those of data 1. The *square sine* with a 90° phase shift window function was manipulated. **c** Data flow of the evaluation. In order to randomize the shape of the wiggles of signal 2 at the position of signal 1, which may affect the peak intensity, the calculation was repeated with random changes of the chemical shift of signal 2 within the range of ±15 Hz. In order to mimic the practical conditions, the phase of the data before the insertion was randomly set in the range of ±7.5°, and in the cases with ANAFOR and SIFT, the misinformation in the chemical shifts of signal 2 was randomly set in the range of ± 3 Hz. **d**, **e** Interferograms in the cases of 100 % (**d**) and 25 % (**e**) sampling coverages. **f** Plots of the root mean squares of the differences of the peak height ratios and the averaged peak height ratios under the conditions with 128 point data without reconstruction, RMSΔ(*h*_(ii)_/*h*_(i)_), of the spectra reconstructed from the synthetic data

It is possible that the sensitivity problem in dilute samples could be alleviated by the observation of the truncated time-domain data with a large number of scans and the insertion of the calculated data with long evolution times (Led and Gesmar 1991). To evaluate whether the sensitivity enhancement can be achieved by the reconstruction, we created synthetic data, and by using the data, we compared the sensitivities of the spectra reconstructed by LP, SIFT, ANAFOR, and Co-ANAFOR. CS was not included, because it is difficult to precisely compare the spectra reconstructed from non-uniformly sampled data by CS with those reconstructed from truncated uniformly sampled data by the aforementioned methods. Two one-dimensional data, which mimic the typical interferograms of ^1^H–^15^N HSQC spectra of proteins, were considered, as shown in Fig. 2a, b (See Electronic Supplementary Material for details). Spectra with 128 points were reconstructed from 16 to 128 point truncated time-domain data by LP, SIFT, ANAFOR, or Co-ANAFOR, and the peak height ratios of signal 1, h_(ii)_/h_(i)_, were calculated (Fig. 2c). The calculation was repeated with random changes of a noise pattern, and the root mean squares of the differences between h_(ii)_/h_(i)_ of the reconstructed spectra and the averaged h_(ii)_/h_(i)_ under the conditions with 128 point data without the reconstruction, which are referred to as RMSΔ(h_(ii)_/h_(i)_), were plotted.

In order to mimic the observation of the truncated time-domain data with a large number of scans, the S/N ratios of the data before the insertion were set to be inversely proportional to the square root of the sampling coverage (Fig. 2d, e). Therefore, the sensitivity enhancements are indicated by the decrease of RMSΔ(h_(ii)_/h_(i)_) with the decrease of the sampling coverage.

The RMSΔ(h_(ii)_/h_(i)_)s of the spectra reconstructed by LP, SIFT, ANAFOR, and Co-ANAFOR at the various sampling coverages are shown in Fig. 2f. In the cases with >70 % sampling coverage, the RMSΔ(h_(ii)_/h_(i)_)s obtained for LP, SIFT, ANAFOR, and Co-ANAFOR decreased with the decrease of the sampling coverage, suggesting that the sensitivity enhancement was achieved by the reconstruction. The RMSΔ(h_(ii)_/h_(i)_)s of the spectra reconstructed by ANAFOR were significantly higher than those reconstructed by the other methods, in the cases with >60 % sampling coverage. We could observe this artifact in the spectra of EIN reconstructed by ANAFOR with 75 % sampling coverage (Fig. 1h). In the cases with <70 % sampling coverage, the RMSΔ(h_(ii)_/h_(i)_)s of the spectra reconstructed by LP and SIFT increased with the decrease of the sampling coverage. These results are in agreement with the low resolution of the EIN spectra reconstructed by LP or SIFT with 25 % sampling coverage (Fig. 1e, g). In contrast, the RMSΔ(h_(ii)_/h_(i)_)s of the spectra reconstructed by Co-ANAFOR were decreased with the decrease of sampling coverage until ~50 % sampling coverage, suggesting that further sensitivity enhancement can be achieved using Co-ANAFOR under these conditions.

**Fig. 3:**
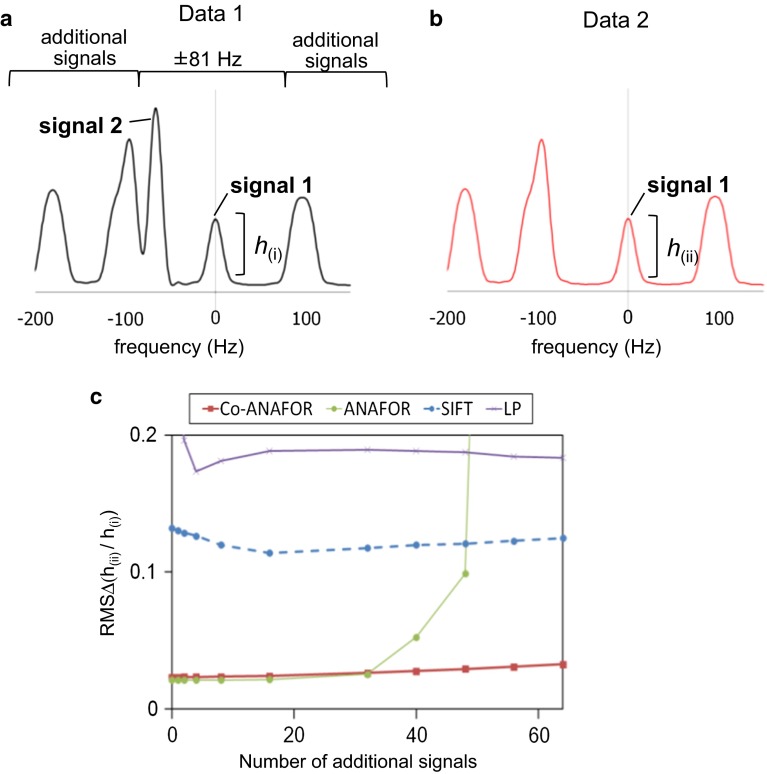
Evaluation of the reconstruction methods using synthetic data with additional signals. **a**, **b** Overlay of the spectra of data 1 (**a**) and data 2 (**b**) in the 128-point synthetic data without reconstruction. The chemical shifts, linewidths, and volumes of signals 1 and 2 are equal to those in Fig. 2. In order to estimate the possibility that the position and distribution of the additional signals may be accidentally advantageous for certain methods, the calculation was repeated with various distributions of the additional signals, by randomly changing their chemical shifts. The *square sine* with a 90° phase shift window function was manipulated. **c** Plots of RMSΔ(*h*_(ii)_/*h*_(i)_) values of the spectra reconstructed from synthetic data with the additional signals

In order to evaluate the accuracy of the peak height ratios of the signals in the crowded spectra reconstructed by LP, SIFT, ANAFOR, and Co-ANAFOR, other synthetic data with various number of signals were created, as shown in Fig. 3a, b, and the RMSΔ(h_(ii)_/h_(i)_)s were calculated for the spectra reconstructed by these methods at 25 % sampling coverage (Fig. 3c). As a result, the RMSΔ(h_(ii)_/h_(i)_)s of the spectra reconstructed by ANAFOR increased with the increase in the number of additional signals (Fig. 3c). We could observe these artifacts as the strong artifacts in the crowded regions of the EIN spectra reconstructed by ANAFOR (Fig. 1a, i). In contrast, the RMSΔ(h_(ii)_/h_(i)_)s of the spectra reconstructed by Co-ANAFOR were almost independent of the number of additional signals (Fig. 3c).

Further analyses using the synthetic data revealed that the effects of dynamic range of intensities, noise, and misinformation in relaxation rates on the reconstruction by Co-ANAFOR are small (See Electronic Supplementary Material for details).

### Application of Co-ANAFOR to the TCS experiments between a membrane protein and its ligand protein

**Fig. 4:**
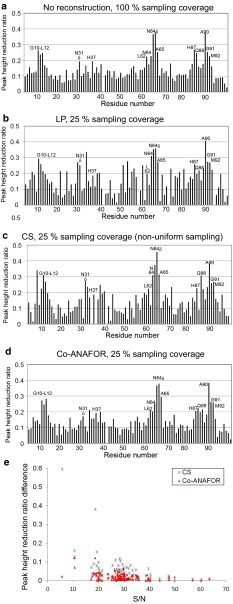
TCS experiments with an excess amount of Pc relative to the photosystem I and cytochrome *b*_*6*_ *f* embedded in thylakoid vesicles. **a**–**d** Plots of the reduction ratios of the signal intensities originating from the amide groups, with and without presaturation, **a** is derived from the 56-point spectra without reconstruction, and **b** is derived from 14-point experimentally observed data and 42-point data inserted for reconstruction by LP, **c** is derived from 14-point non-uniformly sampled data and 42-point data inserted for reconstruction by CS, and **d** is derived from 14-point experimentally observed data and 42-point data inserted for reconstruction by Co-ANAFOR. The residues with >0.2 signal intensity reduction ratios in the previously reported 56-point unreconstructed data and 56-points inserted for reconstruction by LP (Ueda et al. 2012) are labeled. **e** Plots of the difference between (**c**) and (**a**) (*black*) and that between (**d**) and (**a**) (*red*) against the signal intensities relative to the root mean squares of the noise level

In order to examine the accuracy of the signal intensity ratios in NMR spectra reconstructed from experimentally observed data by Co-ANAFOR, we applied Co-ANAFOR to the previously reported data of TCS experiments with excess amounts of [ul-^2^H,^15^N] spinach plastocyanin relative to non-labeled photosystem I and cytochrome *b*_*6*_ *f* embedded in thylakoid vesicles, in which LP was utilized for the reconstruction (Fig. 4; Electronic Supplementary Material Fig. S5) (Ueda et al. 2012). The lineshapes of the signals in the 56-point spectra without reconstruction (Fig. 4a; Electronic Supplementary Material Fig. S5a) were significantly different from those in the spectra from the 14-point uniformly sampled data and the 42-point data inserted for reconstruction by LP (Fig. 4b; Electronic Supplementary Material Fig. S5b). Therefore, 28-point experimentally recorded data were required to obtain 56-point spectra without signal distortion, in the case of LP (Electronic Supplementary Material Fig. S5c). Although severe signal distortion was not observed for the spectra from the 14-point non-uniformly sampled data and the 42-point data inserted for reconstruction by CS (Electronic Supplementary Material Fig. S5d), the peak height reduction ratios of the relatively weak signals with signal-to-noise ratio (S/N) <40 were remarkably different from those of the 56-point data without reconstruction (Fig. 4c, e). In contrast, the spectra from the 14-point uniformly sampled data and the 42-point data inserted for reconstruction by Co-ANAFOR were similar to that from the 56-point spectra without reconstruction (Electronic Supplementary Material Fig. S5e), and the differences of the peak height ratios between them were <0.15 for all signals (Fig. 4d, e).

## Discussion

We developed an interferogram reconstruction method, Co-ANAFOR, in which the previously reported ANAFOR method (Bodart et al. 2002; Lippens et al. 2003) was improved as follows. Firstly, the Tikhonov regularization was applied in the least squares calculation. Secondly, the experimentally recorded data were retained, and the calculated data were inserted exclusively into the unobserved data point region.

The reconstruction of both synthetic and experimentally observed data revealed that the accuracy of the spectra reconstructed by ANAFOR is higher than those of the spectra reconstructed by conventional LP and SIFT, under conditions with low sampling coverages (Figs. 1a, e, g, i, 2f). This is due to the direct utilization of the chemical shifts determined by the guide-FIDs in ANAFOR. However, the accuracy of the reconstruction by ANAFOR was low in the crowded spectra (Figs. 1i, 3c) and/or in the presence of misinformation between the truncated data and the guide-FIDs (Figs. 1h, 2f). These vulnerabilities of ANAFOR were rescued by the improvements, mentioned above, in Co-ANAFOR (Figs. 1l, m, 2f, 3c).

The analyses using the synthetic data revealed that the accuracy of the peak height ratios can be increased with the increase of the number of scans of each sampling point and the insertion of the calculated data with a long evolution time by Co-ANAFOR (Fig. 2), In addition, the accuracy of the peak height reduction ratios of the spectra reconstructed from truncated time-domain data by Co-ANAFOR was higher than those reconstructed from non-uniformly sampled data by CS, under conditions where the sampling coverages were identical and the reconstructed spectra exhibited similar resolutions, in the TCS experiments of plastocyanin and photosystem I and cytochrome *b*_*6*_ *f* embedded in thylakoid vesicles (Fig. 4; Electronic Supplementary Material Fig. S5). Therefore, improvements in the accuracy of the signal intensity ratios can be achieved by Co-ANAFOR.

In the TCS experiments of plastocyanin and photosystem I and cytochrome *b*_*6*_ *f* embedded in thylakoid vesicles, the residues with signal intensity reduction ratios >0.2 of the spectra reconstructed by Co-ANAFOR were almost identical to those of the unreconstructed spectra (Fig. 4). Therefore, as reported previously (Ueda et al. 2012), we could conclude that the hydrophobic patch residues of plastocyanin are in close proximity to photosystem I, whereas the acidic patch residues of plastocyanin do not form stable salt bridges with either photosystem I or cytochrome *b*_*6*_ *f*, in the electron transfer complexes.

Co-ANAFOR, as well as ANAFOR and SIFT, requires knowledge of peak positions, although the knowledge is not required in the reconstruction of the non-uniformly sampled data by CS. Therefore, Co-ANAFOR is not applicable to experiments for the determination of chemical shifts. In addition, Co-ANAFOR requires additional measurements of the guide-FIDs, in contrast to the conventional methods with LP, and optimization of the following variable parameters, which can affect the peak height ratios in the reconstructed spectra, is required for Co-ANAFOR: (1) the transverse relaxation rates utilized for the calculation of the inserted data, (2) the linewidths of the signals in the directly observed dimension, and (3) the Thikohov regularization factor. Therefore, in comparison with LP and CS, additional measurements and processing time are required for the reconstruction by Co-ANAFOR, as well as by ANAFOR and SIFT. However, the guide-FIDs can be usually measured in a short time, because the guide-FIDs experiments are sensitive, due to their simple coherence transfer pathways. In addition, the default values are empirically widely applicable for the parameters described above. Therefore, only a small amount of additional time is required for the measurement and processing using Co-ANAFOR.

Co-ANAFOR is effective without using non-uniform sampling, because the collection of sampling points with a long evolution time is avoided in Co-ANAFOR, by the utilization of the chemical shifts of the signals in the guide-FIDs. Although only the uniformly sampled data were processed here, Co-ANAFOR can also be applied to non-uniformly sampled data. Reconstruction of the spectra from the non-uniformly sampled data by Co-ANAFOR may be effective, particularly under conditions where the transverse relaxation during the evolution time is negligible, such as in the constant time evolution domain.

The application of the Tikhonov regularization results in a subtle but significant reduction of the signal intensities, and the misinformation in the transverse relaxation rates, which is due to the utilization of the uniform transverse relaxation rates for the calculation of the inserted data, can cause signal intensity errors, although these errors are much smaller than those in ANAFOR (Fig. 1h, l, 2f). Therefore, Co-ANAFOR may not be suitable for cases where precise absolute signal intensities are required, although these effects are largely cancelled out with the calculations of the peak height ratios. Although Co-ANAFOR is primarily designed to determine the peak heights in two-dimensional spectra relative to those in another spectrum, it is also applicable to the reconstruction of more than two dimensional spectra and the spectra in chemical shift perturbation experiments (See Electronic Supplementary Material).

Regardless of the reconstruction methods, optimization of both the window function parameters and the maximum evolution times after the insertion is practically important. However, this is not a simple task, because the number and distribution of signals in each interferogram of multidimensional NMR data of proteins are different, and the transverse relaxation rates of each signal are also different. We empirically recommend the following conditions for the reconstruction of two-dimensional spectra of proteins: the maximum evolution time after the insertion and the sampling coverage are ~120 % of the averaged transverse relaxation time and ~30 %, respectively, and the window function is a monotonic decreasing function, such as a square sine with a 90° phase shift. The recommended evolution time after the insertion is identical to those for other reconstruction methods (Levitt et al. 1984; Rovnyak et al. 2004). These parameters are optimized by performing simulations using synthetic data that are similar to the experimental data.

For accurate determination of the signal intensity ratios from the spectra reconstructed by Co-ANAFOR, the chemical shifts of the guide-FIDs should be identical to those of the truncated data. Therefore, the guide-FIDs are usually recorded before or after the truncated data measurements at the same temperature. The resolution of the guide-FIDs in the dimension for reconstruction should be equal to or larger than that of the reconstructed spectra. We can notice large misinformation in the chemical shifts as the differences in lineshapes between the spectra reconstructed by Co-ANAFOR and those from guide-FIDs. For example, in the red spectrum shown in Electronic Supplementary Material Fig. S7b, which was reconstructed by Co-ANAFOR with the black spectrum utilized as the guide-FIDs, the signals with different chemical shifts from those of the guide-FIDs, which are perturbed upon addition of tetralysine, were observed as broad signals.

Accumulating evidences have suggested that proteins are quite dynamic and interconvert between multiple conformations in equilibria (Jeschke 2012; Brucale et al. 2014). NMR methods, such as residual dipolar couplings (Tjandra and Bax 1997; Tolman and Ruan 2006; Ban et al. 2013), relaxation dispersion (Palmer 2004; Boehr et al. 2006; Baldwin and Kay 2009) and paramagnetic relaxation enhancement (Iwahara and Clore 2006; Tang et al. 2006; Clore and Iwahara 2009; Clore 2014) experiments, are useful for obtaining information about the conformational dynamics of proteins. Application of Co-ANAFOR to these experiments would enable analyses for targets with low yields and stabilities.

## Electronic supplementary material

Below is the link to the electronic supplementary material.

Supplementary material 1 (DOCX 1022 kb)
