# Supplementary material for: Development of a method for reconstruction of crowded NMR spectra from undersampled time-domain data
Source: J Biomol NMR. 2015 Feb 13;62(1):31–41. doi: 10.1007/s10858-015-9908-9 (PMC4432090; doi:10.1007/s10858-015-9908-9)
Supplement: Supplementary file 1 — Supplementary material 1 (DOCX 1022 kb) [file 10858_2015_9908_MOESM1_ESM.docx]

Supplemental material to: **“Development of a method for reconstruction of crowded NMR spectra from undersampled time-domain data”**

Takumi Ueda, Chie Yoshiura, Masahiko Matsumoto, Yutaka Kofuku, Junya Okude, Keita Kondo, Yutaro Shiraishi, Koh Takeuchi, and Ichio Shimada

**Table of Contents**

**SI text.**

· Principles of ANAFOR and the developed method.

· Details of the processing of the synthetic data.

· Evaluation of the effect of the dynamic range, noise, and misinformation using synthetic data.

· Additional applications of Co-ANAFOR.

**Fig. S1.** Data flow of the developed method.

**Fig. S2.** Evaluation of the reconstruction methods using synthetic data with various intensities of the additional signals.

**Fig. S3.** Evaluation of the reconstruction methods using synthetic data with various noise levels.

**Fig. S4.** RMS*Δ*(*h*_(ii)_/*h*_(i)_) values of the spectra reconstructed by Co-ANAFOR from the synthetic data with mismatches in chemical shifts, linewidth, or phases.

**Fig. S5.** Evaluation of Co-ANAFOR using the experimentally observed TCS data at 18.8 T, for an excess amount of [ul-^2^H, ^15^N] plastocyanin relative to the photosystem I and cytochrome *b_6_f* embedded in thylakoid vesicles.

**Fig. S6.** Application of Co-ANAFOR to the 3D ^15^N-edited NOESY experiments.

**Fig. S7.** Application of Co-ANAFOR to the chemical shift perturbation experiments.

**SI Text**

**Principles of ANAFOR and the developed method**

Although ANAFOR and the method developed in this manuscript are theoretically applicable to more than two-dimensional NMR (Bodart et al., 2002; Lippens et al., 2003), we will first consider the processing of two-dimensional NMR data, for simplicity. Interferograms are generated by processing of the directly observed dimension of two-dimensional NMR data. Under conditions where the phase is correctly adjusted and the signals are composed of decaying sinusoids, with chemical shifts and transverse relaxation times identical to those of the guide FIDs, an interferogram *F*(*t*), which is composed of its real part *f*_r_(*t*) and imaginary part *f*_i_(*t*), can be represented as a linear combination of *k* signals in the guide FIDs (Bodart et al., 2002; Lippens et al., 2003): $F\left( t \right)=f_{r}\left( t \right)+f_{i}\left( t \right)=\sum_{m=1}^{k} x_{m}exp(-R_{m}+i\left( \omega_{m}-\omega_{0} \right)t)$ (1)

where *x*_m_, *ω*_m_, and *R*_m_ are the intensity, frequency, and transverse relaxation rates of the *m*th signal, respectively, and *ω*_0_ is the carrier frequency. The substitution of *ω*_m_ and *R*_m_ for the chemical shifts and transverse relaxation rates determined by the guide-FIDs, respectively, and the substitution of *f*_r_(*t*) and *f*_i_(*t*) matrices in eq.1 for the truncated time domain data result in the following equations, where the numbers of equations and unknown parameters are the number of the sampling points in the indirect dimension and *k*, respectively:

$\left( \begin{matrix} A_{r}(1,t_{0}) & \cdots& A_{r}(k,t_{0}) \\ A_{r}(1,t_{0}+\Delta t) & \cdots& A_{r}(k,t_{0}+\Delta t) \\ \vdots& \ddots& \vdots\\ A_{r}(1,t_{amax}) & \cdots& A_{r}(k,t_{amax}) \\ A_{i}(1,t_{0}) & \cdots& A_{i}(k,t_{0}) \\ A_{i}(1,t_{0}+\Delta t) & \cdots& A_{i}(k,t_{0}+\Delta t) \\ \vdots& \ddots& \vdots\\ A_{i}(1,t_{amax}) & \cdots& A_{i}(k,t_{amax}) \end{matrix} \right)\left( \begin{matrix} x_{1} \\ x_{2} \\ \vdots\\ x_{k} \end{matrix} \right)=\left( \begin{matrix} f_{r}(t_{0}) \\ f_{r}(t_{0}+\Delta t) \\ \vdots\\ f_{r}(t_{amax}) \\ f_{i}(t_{0}) \\ f_{i}(t_{0}+\Delta t) \\ \vdots\\ f_{i}(t_{amax}) \end{matrix} \right)$ (2)

where *t*_0_ and *t*_amax_ are shortest and longest evolution times in the indirect dimension, respectively, and *Δt* is the dwell time. The optimal solutions of *x*_m_ can be calculated by the least squares method or its variants.

In the case of ANAFOR, the optimal solution of ***x*** is provided by the standard least-squares method, as follows:

$\mathbf{x}={(\boldsymbol{A}^{T}\boldsymbol{A})}^{-1}\boldsymbol{A}^{T}\boldsymbol{F}$ (3)

where ***A***, ***x***, and ***F*** are the first, second, and third matrices in eq. (2). In the case of the developed method, the optimal solution of ***x*** is provided by the Tikhonov regularization (or ridge regression) method, as follows:

$\mathbf{x}={(\boldsymbol{A}^{T}\boldsymbol{A+}\lambda\boldsymbol{I})}^{-1}\boldsymbol{A}^{T}\boldsymbol{F}$ (4)

where ***I*** is the identity matrix and *λ* is the Tikhonov regularization factor*.* In the standard least-squares and the Tikhonov regularization, $\left\| \boldsymbol{Ax}-\boldsymbol{F} \right\|^{2}$ and $\left\| \boldsymbol{Ax}-\boldsymbol{F} \right\|^{2}+\left\| \lambda\boldsymbol{I} \right\|^{2}$ are minimized respectively.

In the case of *λ*=0, eq. (4) is identical to eq. (3). In the cases of large numbers of signals, relative to the number of truncated time-domain data, the solutions of the least square equations are stabilized with the increase of *λ*. The increase of *λ* also causes a decrease of the signal intensities, and **x** approaches a zero vector as *λ* tends to infinity. *λ* is empirically set to 0.01, to stabilize the solutions without severely decreasing the signal intensities.

The calculation of the unobserved data is accomplished by the substitution of *x*_m_ in eq.(1) for the solution of ***x***. In ANAFOR, the original truncated time-domain data are replaced with the calculated data. In the developed method, only the unrecorded data with large evolution times were inserted. The time-domain data after the insertion are transformed with the fast Fourier transform (FFT) algorithm.

Although the transverse relaxation rates of each signal in the guide-FIDs are required for the reconstruction by ANAFOR, the determination of these rates is time-consuming. In this report, they were uniformly set to the reciprocal of the maximum evolution time after the insertion. Therefore, in practice, the transverse relaxation rates utilized for the calculation of the inserted data are not always identical to those of the truncated time-domain data.

The dataflow of the developed method is shown in Electronic Supplementary Material Fig. S1. The truncated time-domain data and the guide-FIDs are processed by standard NMR data processing software, and the resulting chemical shift tables of the guide-FIDs and the low resolution spectrum are exported to the developed program. The low resolution spectrum is transformed with the inverse fast Fourier transform algorithm, and matrices ***A*** and ***F*** are generated. The optimal solution of ***x*** is calculated, and the solution is utilized for the insertion of the unrecorded data. The time domain data are manipulated with zero-filling and window functions, and then are transformed with the FFT algorithm. The reconstructed high resolution spectrum is exported to standard NMR data processing software for visualization. The run-time of the developed program for the 2D ^1^H-^15^N HSQC-type data of proteins was less than one minute with a standard personal computer.

Linear Prediction Singular Value Decomposition (LPSVD) (Barkhuijsen et al., 1985) and filter diagonalization (Chen et al., 2000; Mandelshtam, 2001; Celik et al., 2012) are also based on the eq. (1). In the case of LPSVD and filter diagonalization, the information about the number of signals and their chemical shifts is directly extracted from the time domain signals, whereas the chemical shifts determined by guide-FIDs are utilized in the case of Co-ANAFOR, as well as ANAFOR (Bodart et al., 2002; Lippens et al., 2003) and SIFT (Matsuki et al., 2011).

**Details of the processing of the synthetic data**

The calculations of the synthetic were performed by in-house developed programs written in a programming language, Python 2.7, supplemented with extension modules, Numpy 1.5, Scipy 0.9, and l1l2py (<http://slipguru.disi.unige.it/Software/L1L2Py/>), unless otherwise stated. The linear prediction package was derived from nmrglue (J. J. Helmus and C.P. Jaroniec, http://code.google.com/p/nmrglue, The Ohio State University) (Helmus and Jaroniec, 2013). The programs were run on 16 Intel Xeon X5687 3.60 GHz CPUs operating under CentOS 5.8.

In the synthetic data, signals 1 and 2 were separated from each other by 66 Hz. The transverse relaxation rates of signals 1 and 2 were 20 and 5 s^-1^, respectively, and the peak volume of signal 2 was twice that of signal 1. The spectral width and the number of complex sampling points after the insertion were set to 1,920 Hz and 128, respectively, to mimic the typical interferograms of ^1^H-^15^N HSQC spectra of proteins. The time domain data after the insertion of the calculated data were manipulated with a sine-squared window function with a 90° phase shift and zero-filling to 1,024 points. In ANAFOR and Co-ANAFOR, the relaxation rates of signals 1 and 2 in the guide-FIDs were set to 15 s^-1^, and the Tikhonov regularization factor in Co-ANAFOR was set to 0.01. The number of coefficients in LP was set to 8. The width of the bright region in SIFT was set to 54 Hz, and the iteration was repeated 50 times.

In the reconstruction of the synthetic data with noise, the noise generated by Mersenne Twister algorism was added to the truncated time domain data. The ratio of the maximum intensities of the noise and the interferogram at 100% sampling coverage was set to 1:5, and the S/N ratios of the truncated time-domain data were set to be inversely proportional to the square root of the sampling coverage. The phase of the truncated time-domain data was randomly set in the range of ±7.5 degrees, and in the cases with ANAFOR, SIFT, and Co-ANAFOR, the misinformation in the chemical shifts of signal 2 was randomly set in the range of ±3 Hz.

The aforementioned calculations were repeated 512 times with random changes of the chemical shift of signal 2 within the range of 66 ± 15 Hz, in order to randomize the shape of the wiggles of signal 2 at the position of signal 1, which may affect the peak intensity. In addition, the noise, the misinformation, and the chemical shifts of additional signals were re-calculated in each calculation.

In the cases of the synthetic data with additional signals, the chemical shift of each additional signal was randomly set in the range of ± 81-960 Hz. The transverse relaxation rates of the additional signals were set to 15 s^-1^, and the number of additional signals was set to 0, 1, 2, 4, 8, 16, 32, 40, 48, 56, or 64. The chemical shifts and the transverse relaxation rates of the additional signals in the guide-FIDs were identical to those in the truncated data, and the misinformation in the chemical shifts of signal 2 and the phase was not introduced.

**Evaluation of the effect of the dynamic range, noise, and misinformation using synthetic data**

In order to evaluate the effect of the dynamic range of signal intensities, synthetic data that contain signals with various intensities, in addition to signals 1 and 2, were further created, as shown in Electronic Supplementary Material Fig. S2a and b, and RMSΔ(h_(ii)_/h_(i)_)s were calculated for the spectra reconstructed by LP, ANAFOR, SIFT, and Co-ANAFOR at 50% sampling coverage. The chemical shift of the additional signal was randomly set in the range of ± 600-960 Hz, and the other parameters were identical to those in the reconstruction of the synthetic data with additional signals (Fig. 3). As a result, the RMSΔ(h_(ii)_/h_(i)_)s were not significantly affected even in the cases with the signal intensity of the additional signal 64 times larger than signal 1 (Electronic Supplementary Material Fig. S2c). In addition, in order to evaluate the effect of noise, synthetic data with various S/N ratios were created, and RMSΔ(h_(ii)_/h_(i)_)s were calculated for the spectra reconstructed by LP, ANAFOR, SIFT, and Co-ANAFOR at 50% sampling coverage (Electronic Supplementary Material Fig. S3). The ratio of the maximum intensities of noise and the interferogram was set to 1:20, 1:15, 1:10, 1:7.5, 1:5, 1:3.75, 1:2.5, 1:1.75, and 1:1.25, and the other parameters were identical to those in the reconstruction of the synthetic data with noise (Fig. 2). As a result, the pattern of the increase of the RMSΔ(h_(ii)_/h_(i)_)s with the decrease of the S/N ratios were similar to those of the unreconstructed data. These results suggest that Co-ANAFOR is applicable to the data with high noise level and dynamic range.

To further evaluate the effects of misinformation in the chemical shifts, phases, and relaxation rates on the peak height ratios in the spectra reconstructed by Co-ANAFOR, RMSΔ(h_(ii)_/h_(i)_)s were calculated using the synthetic data in the presence of each types of misinformation (Electronic Supplementary Material Fig. S4). The effects of the misinformation of relaxation ratios on the RMSΔ(h_(ii)_/h_(i)_)s were relatively small (Electronic Supplementary Material Fig. S4a and b), and the RMSΔ(h_(ii)_/h_(i)_) values were < 0.1 in the cases with uniform values applied as the transverse relaxation rates of signals 1 and 2 in the guide-FIDs (Electronic Supplementary Material Fig. S4c).

**Additional applications of Co-ANAFOR**

Although Co-ANAFOR is primarily designed to determine the peak heights in two-dimensional spectra relative to those in another spectrum, Co-ANAFOR is also applicable to more than two-dimensional spectra. The F1 (^1^H)-F2 (^15^N) slices of the 3D ^15^N-edited NOESY spectra of ubiquitin reconstructed by Co-ANAFOR or CS with 12.5 % sampling coverage were similar to those from the unreconstructed spectra (Electronic Supplementary Material Fig. S6).

Co-ANAFOR is also applicable to the chemical shift perturbation experiments (Electronic Supplementary Material Fig. S7). In the ^1^H-^15^N HSQC spectra of [u-^15^N]plastocyanin-tetralysine complex reconstructed by Co-ANAFOR, in which the spectrum of the unbound plastocyanin was utilized as the guide-FIDs, the signals with different chemical shifts from those of the guide-FIDs can be observed as broad signals (Electronic Supplementary Material Fig. S7b), such as those in the spectra reconstructed by zero-filling (Electronic Supplementary Material Fig. S7c), because only noise is inserted for the truncated interferogram of these signals. In addition, the signals with chemical shifts that are almost identical to the guide-FIDs are observed with lineshapes similar to those in the unreconstructed spectra (Electronic Supplementary Material Fig. S7a and b), and thus the resolutions of the spectra reconstructed by Co-ANAFOR are higher than those of spectra reconstructed by zero-filling (Electronic Supplementary Material Fig. S7b and c).


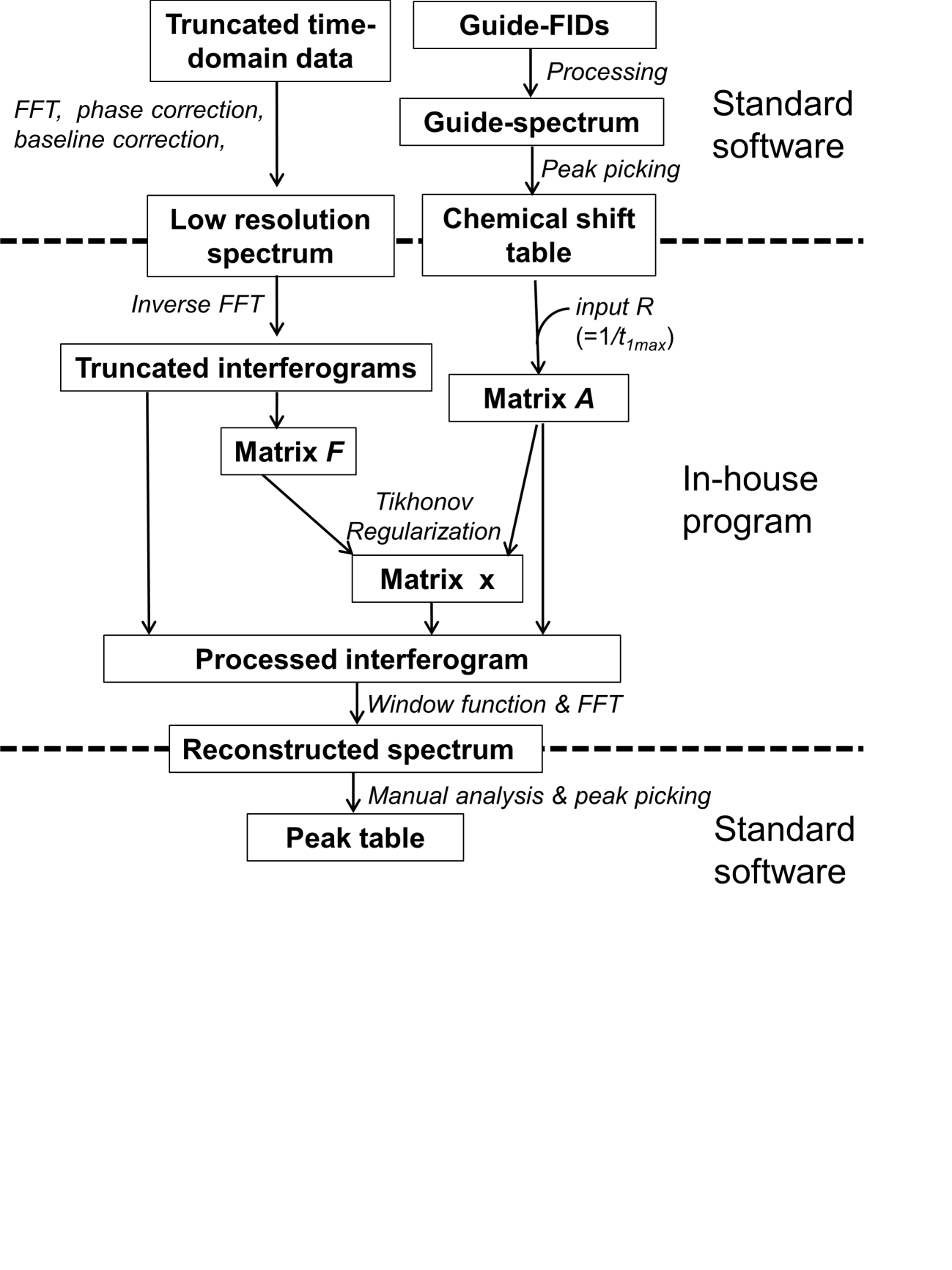


**Fig. S1. Data flow of the developed method.**

**
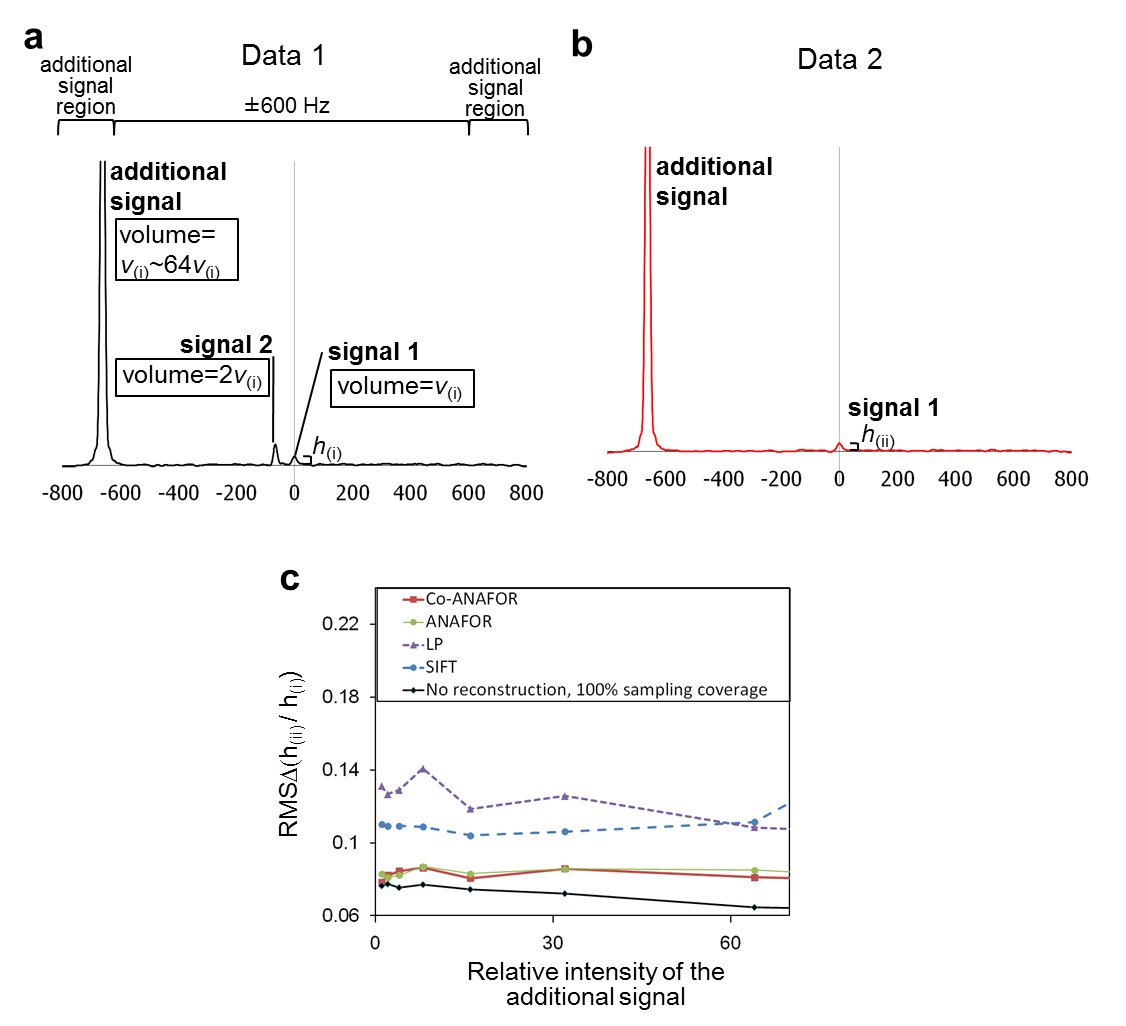
Fig. S2. Evaluation of the reconstruction methods using synthetic data with various intensities of the additional signals.**

(a) and (b). Overlay of the spectra of data 1 (a) and data 2 (b) in the 128-point synthetic data without reconstruction. The chemical shifts, linewidths, and volumes of signals 1 and 2 are equal to those in Fig. 3. The calculation was repeated with various distributions of the additional signals, by randomly changing their chemical shifts. The square sine with a 90° phase shift window function was manipulated. (c). Plots of RMS*Δ*(*h*_(ii)_/*h*_(i)_) values of the spectra reconstructed from synthetic data with the additional signals.

**
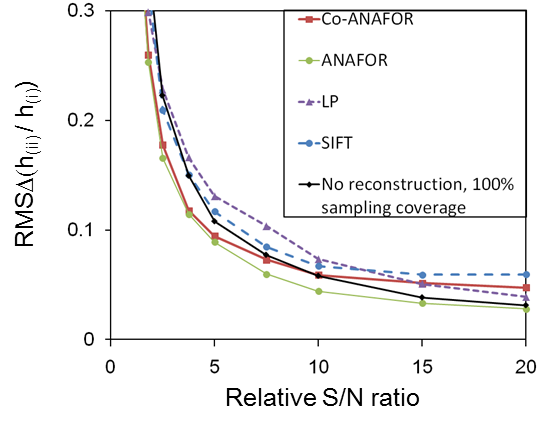
**

**Fig. S3. Evaluation of the reconstruction methods using synthetic data with various noise levels.**

Plots of RMS*Δ*(*h*_(ii)_/*h*_(i)_) values of the spectra reconstructed from the synthetic data shown in Fig. 2, with the modification of the noise level, at 50% sampling coverage, and those of the unreconstructed spectra.

.

**
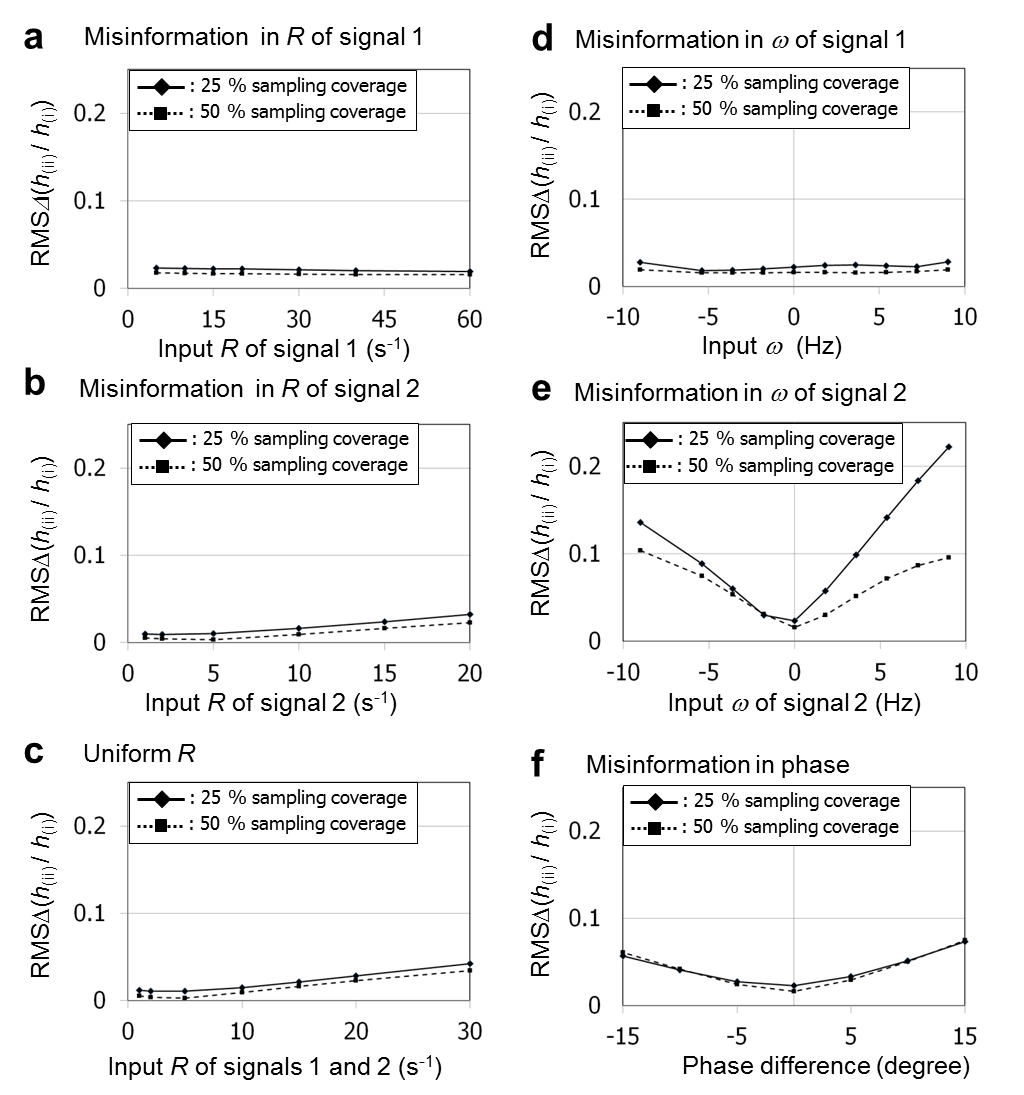
**

**Fig. S4. RMS*Δ*(*h*_(ii)_/*h*_(i)_) values of the spectra reconstructed by Co-ANAFOR from the synthetic data with mismatches in chemical shifts, linewidth, or phases.**

(a) and (b). Plots of RMS*Δ*(*h*_(ii)_/*h*_(i)_) values in the presence of the misinformation in the transverse relaxation rates (*R*) of signal 1 (a) or signal 2 (b). (c). Plots of RMS*Δ*(*h*_(ii)_/*h*_(i)_) in the cases with uniform transverse relaxation rates of signals 1 and 2 utilized for the calculation of the inserted data. (d) and (e). Plots of RMS*Δ*(*h*_(ii)_/*h*_(i)_) values in the presence of the misinformation in the chemical shifts (*ω*) of (d) signal 1 or (e) signal 2. (f). Plots of RMS*Δ*(*h*_(ii)_/*h*_(i)_) values with errors in the phase of the truncated time-domain data. The data with 25% and 50% sampling coverages are represented by solid and dotted lines, respectively. The number of data points after the insertion of the calculated data was 128.

**
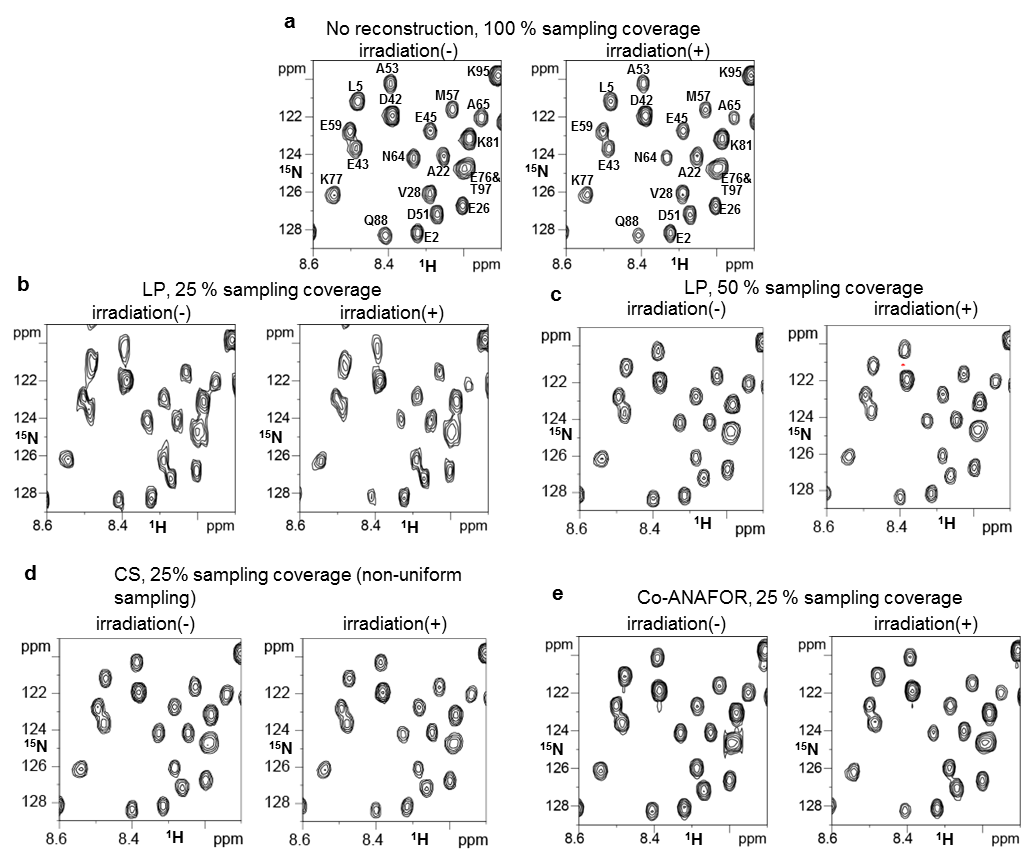
**

**Fig. S5. Evaluation of Co-ANAFOR using the experimentally observed TCS data at 18.8 T, for an excess amount of [ul-^2^H, ^15^N] plastocyanin relative to the photosystem I and cytochrome *b_6_f* embedded in thylakoid vesicles.**  (a). 56-point spectra without reconstruction. (b). Spectra from 14-point experimentally observed data and 42-point data inserted for reconstruction by LP. (c). Spectra from 28-point experimentally observed data and 28-point data inserted for reconstruction by LP. (d). Spectra from 14-point non-uniformly sampled experimentally observed data and 42-point data inserted for reconstruction by CS. (e). Spectra from 14-point experimentally observed data and 42-point data inserted for reconstruction by Co-ANAFOR. For lineshape clarity, the signals are not labeled in b-e.


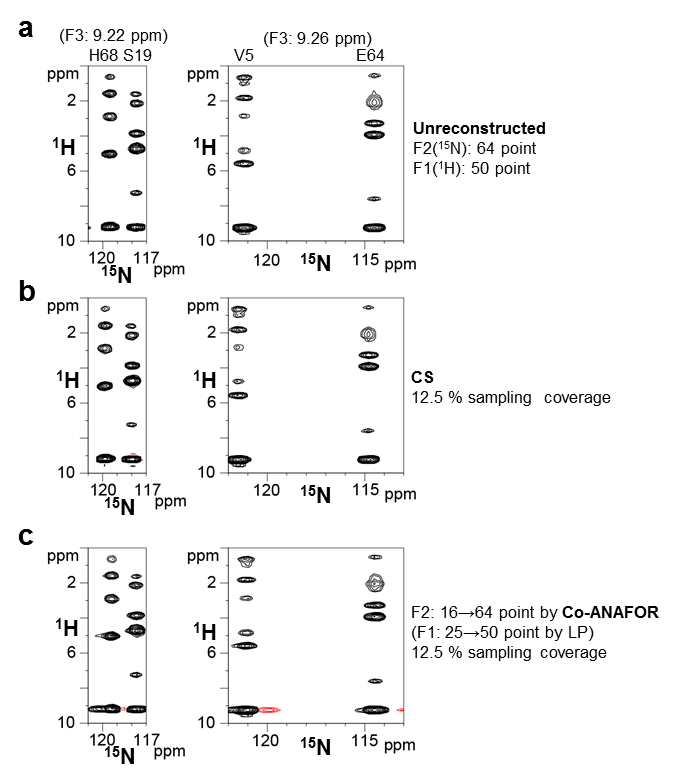


**Fig. S6. Application of Co-ANAFOR to the 3D ^15^N-edited NOESY experiments.**

(a). ^1^H (F1)-^15^N (F2) slices of ^15^N-edited NOESY spectra (^1^H (F3): 9.22 or 9.26 ppm) observed at 14.1 T for [ul-^13^C,^15^N] ubiquitin, without reconstruction in the F1 or F2 dimension. (b). The slices from non-uniformly sampled experimentally observed data and data inserted for reconstruction by CS at 12.5 % sampling coverage. (c). The slices from uniformly sampled truncated data and data inserted for reconstruction by LP and Co-ANAFOR in the F1 and F2 dimensions, respectively, with a ^1^H-^15^N HSQC spectrum utilized as the guide-FIDs.

**
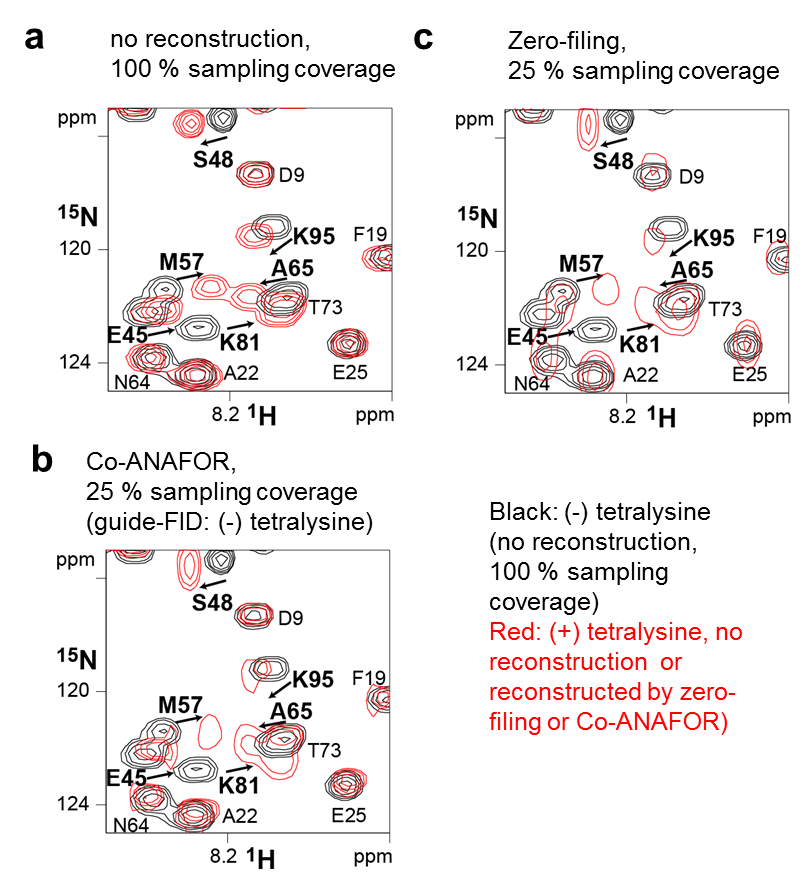
**

**Fig. S7. Application of Co-ANAFOR to the chemical shift perturbation experiments.**

(a). 60-point ^1^H-^15^N HSQC spectra observed at 11.8 T for [ul-^15^N] plastocyanin in the presence (red) or absence (black) of tetralysine, without reconstruction. (b)-(c). Overlay of the 60-point spectra in the absence of tetralysine (black) and those from 15-point experimentally observed data and 45-point data inserted for reconstruction by Co-ANAFOR (b) or 45-point zeros (c) in the presence of tetralysine (red). The data in the absence of tetralysine were utilized as the guide-FIDs. The labels of the resonances that were significantly shifted upon the addition of tetralysine are bold.

**References**

**Barkhuijsen, H., de Beer, R., Bovee, W.M., Creyghton, J.H., and van Ormondt, D.** (1985). Application of linear prediction and singular value decomposition (LPSVD) to determine NMR frequencies and intensities from the FID. Magn. Reson. Med. **2,** 86-89.

**Bodart, P.R., Amoureux, J.P., and Taulelle, F.** (2002). ANAFOR: Application of a restricted linear least squares procedure to NMR data processing. Solid State Nucl. Magn. **21,** 1-20.

**Celik, H., Ridge, C.D., and Shaka, A.J.** (2012). Phase-sensitive spectral estimation by the hybrid filter diagonalization method. J. Magn. Reson. **214,** 15-21.

**Chen, J., Shaka, A.J., and Mandelshtam, V.A.** (2000). RRT: the regularized resolvent transform for high-resolution spectral estimation. J. Magn. Reson. **147,** 129-137.

**Helmus, J.J., and Jaroniec, C.P.** (2013). Nmrglue: an open source Python package for the analysis of multidimensional NMR data. J. Biomol. NMR **55,** 355-367.

**Lippens, G., Bodart, P.R., Taulelle, F., and Amoureux, J.P.** (2003). Restricted linear least square treatment processing of heteronuclear spectra of biomolecules using the ANAFOR strategy. J. Magn. Reson. **161,** 174-182.

**Mandelshtam, V.** (2001). FDM: the filter diagonalization method for data processing in NMR experiments. Prog. Nucl. Mag. Res. Sp. **38,** 159-196.

**Matsuki, Y., Konuma, T., Fujiwara, T., and Sugase, K.** (2011). Boosting protein dynamics studies using quantitative nonuniform sampling NMR spectroscopy. J. Phys. Chem. B **115,** 13740-13745.
